# The role of volume and intensity on the association between physical activity and mental health among adolescents: a cross-sectional study

**DOI:** 10.1590/1984-0462/2023/41/2022010

**Published:** 2023-04-07

**Authors:** Diogo Henrique Constantino Coledam, Gustavo Aires de Arruda, Júlio Wilson dos-Santos, Alessandro Hervaldo Nicolai Ré

**Affiliations:** a Instituto Federal de Educação, Ciência e Tecnologia de São Paulo, Boituva, SP, Brazil.; b Universidade de São Paulo, São Paulo, SP, Brazil.; c Universidade de Pernambuco, Recife, PE, Brazil.; d Universidade Estadual Paulista, Bauru, SP, Brazil.

**Keywords:** Motor activity, Mental disorders, Epidemiology, Exercise, Suicidal ideation, Atividade motora, Desordens mentais, Epidemiologia, Exercício, Ideação suicida

## Abstract

**Objective:**

To analyze the association between volume and intensity of physical activity and mental health among adolescents.

**Methods:**

Cross-sectional study with 604 Brazilian adolescents. Data were assessed using a self-report questionnaire. The outcomes were suicidal ideation, suspicion of common mental disorders, and negative self-perception of mental health. The independent variables were leisure physical activity at low and moderate-to-vigorous intensities. Volume was analyzed in two ways: any volume (presence vs absence), and volume classified according to amount in minutes of weekly physical activity: inactive (0), low active (1–419), and high active (≥420). Poisson regression was performed to estimate prevalence ratios.

**Results:**

Any volume of moderate-to-vigorous physical activity was significantly associated with a lower prevalence ratio of all outcomes (PR 0.67 to 0.77). Compared to inactive adolescents, those who were classified as low active for moderate-to-vigorous intensity, presented a lower likelihood of having suicidal ideation, suspicion of common mental disorders, and negative self-perception of mental health (PR 0.70 to 0.76). Furthermore, high active adolescents in moderate-to-vigorous physical activity presented lower suicidal ideation and negative self-perception of mental health (PR 0.62 and 0.57).

**Conclusions:**

The promotion of moderate-to-vigorous intensity physical activity at any volume can benefit the mental health of adolescents, however, no association was evidenced for low intensity physical activity.

## Resumo

**Objetivo:**

Analisar a associação entre o volume e intensidade da atividade física e a saúde mental de adolescentes.

**Métodos:**

Estudo transversal que envolveu 604 adolescentes brasileiros. Os dados foram coletados por meio de um questionário autorrelatado. Os desfechos foram ideação suicida, suspeita de transtornos mentais comuns e autopercepção negativa de saúde mental. A variável independente foi a atividade física em tempo livre nas intensidades leve e moderada a vigorosa. O volume foi analisado de duas formas: qualquer volume (presença vs. ausência); e de acordo com a quantidade de atividade física semanal em minutos — inativos (0), pouco ativos (1–419) e altamente ativos (≥420). A regressão de Poisson foi realizada para estimar as razões de prevalência (RP).

**Resultados:**

Qualquer volume de atividade física de intensidade moderada a vigorosa foi significativamente associado à menor razão de prevalência de todos os desfechos (RP 0,67 a 0,77). Comparados aos adolescentes inativos, aqueles que foram classificados como pouco ativos na intensidade moderada a vigorosa apresentaram menor probabilidade de ter ideação suicida, suspeita de transtornos mentais comuns e autopercepção negativa de saúde mental (RP 0,70 a 0,76). Da mesma forma, adolescentes altamente ativos na intensidade moderada a vigorosa apresentaram menor ideação suicida e autopercepção negativa de saúde mental (RP 0,62 e 0,57).

**Conclusões:**

A promoção de atividade física moderada a vigorosa em qualquer volume pode beneficiar a saúde mental de adolescentes, no entanto nenhuma associação foi evidenciada para a atividade física na intensidade leve.

## INTRODUCTION

Common mental disorders, represented by depressive and anxiety symptoms, have a multifactorial etiology and are the leading causes of illness and disability among adolescents worldwide.^1^ The early development of these disorders has received great attention because they are associated with comorbidities, substance abuse, social and occupational dysfunction, social exclusion, discrimination, stigma, educational difficulties, health-risk behaviors, physical ill-health, human rights violations, and suicidal ideation and attempt.^1,2^ Furthermore, depression in adolescence is associated with long-term outcomes, such as the recurrence of mental disorders, poor physical health, financial, educational, and social problems, and unemployment.^3,4^

Physical activity (PA) is a behavior associated with a variety of health outcomes among adolescents, including reduced symptoms of depression and anxiety.^5^ Observational studies have demonstrated that PA is associated with a better profile of perceived mental health,^6^ general mental health,^7-9^ depressive symptoms,^6,7,9-12^ anxiety,^6,7,10^ and suicidal ideation and attempt.^13-16^ Although the causality of these connections is still only partially supported by the literature,^17^ the promotion of PA could be a strategy to improve the mental health of adolescents, in addition to prevent other causal risk factors of mental disorder development.^1^

Despite the large amount of information available, there is a lack of evidence regarding the role of volume and intensity in the association between PA and mental health. Investigation of this association will clarify whether the current guideline — an average of 60 minutes/day of moderate-to-vigorous intensity PA^5^ — is the most appropriate target for adolescents, as well as for use in epidemiological studies. Considering that 81% of adolescents worldwide do not meet the current PA guidelines,^18^ it is important to analyze whether other volumes and intensities can also provide mental health benefits and, consequently, be perceived as achievable by inactive youth.^19^ Another contribution is that the information from this study could support the development of specific guidelines for the prevention of mental disorders in young people. In view of this, the aim of the present study was to analyze the association between volume and intensity of PA and mental health among adolescents.

## METHOD

This is a cross-sectional study conducted in 2019 in Boituva city, São Paulo, Brazil. The project was approved by the local Ethics Research Committee of the Federal Institute of Education, Science and Technology of São Paulo (IFSP), São Paulo, Brazil, protocol 06438418.1.0000.5473, on March 12, 2019.

The population for this study was estimated at 2,460 adolescents enrolled in high school, and the city analyzed contains three high schools in the public education system. The sample size was calculated through the following parameters: N=2,460, prevalence of 50%, precision of 5%, design effect of 1.5, and confidence interval of 95%, using the software Open Epi, OpenEpi Project (Atlanta, US), version 3.01. The minimum number of participants required was 499. Data collection was conducted in a probabilistic sample of 675 adolescents and the final was composed of 604 adolescents. The sample size was sufficient to detect significant associations when the difference in the prevalence of the outcome between exposed and non-exposed participants was ≥10%, considering the sample characteristics of ratio of non-exposed to exposed to PA, a power of 80%, and significance of 5%.

All schools were invited to compose the study and two agreed to participate. The inclusion criteria were: be enrolled in a public high school and present no limitation that would prevent performance of leisure PA or the comprehension of the questionnaire. The participants were randomly selected from each classroom, stratified by sex, course, and grade of study.

All procedures took place at the school where the participants were enrolled. The schools were visited by researchers and the project was presented to the principals. After receiving authorization from the principals, the objectives, procedures, risks and benefits of the project were presented to all students in each classroom. Informed assent and consent forms were distributed and after the return of the signed documents, data collection was scheduled. No information that could identify the participant was available in the questionnaire, and after completion, the participant deposited it in an opaque box.

The dependent variables were suspicion of common mental disorders, suicidal ideation, and negative self-perception of mental health. The suspicion of common mental disorders was estimated using the Brazilian version of the Self-Reporting Questionnaire (SRQ-20).^20^ The questionnaire contains 20 questions related to symptoms in the previous 30 days, distributed into four dimensions: depressive-anxious mood, decrease in vital energy, somatic symptoms, and depressive thoughts. The instrument presents acceptable psychometric properties and an internal consistency of 0.80. A previous study with a Brazilian sample proposed a cutoff ≥8 for suspicion of common mental disorder, with sensitivity and specificity of 86.33% and 89.31% respectively, a discriminant power of 0.9, and Cronbach's alpha of 0.86, adopting a psychiatric interview as the reference criterion.^21^ The questionnaire also presented high internal consistency (Cronbach alpha 0.75) and reliability (ICC [Intraclass correlation Coefficient] 0.85) among Brazilian adolescents.^22^ One item on the scale “Do you have difficulty at work? (Does your work cause suffering?)” was adapted for the school context as previously suggested.^22^ Suicidal ideation was assessed using the question “Have you been thinking about ending your life?”, with answer options “Yes” or “No”.^20^ Negative mental health self-perception was estimated using the question “In general, how do you consider your mental health”, with options “good” or “bad”.

The independent variable was PA, assessed using the Brazilian version of the long form of the International Physical Activity Questionnaire (IPAQ).^23^ PA during recreation, sport, exercise, and leisure time was considered, in bouts of 10 min of continuous activity. Data were stratified into PA intensity at low physical activity (LPA), and moderate-to-vigorous physical activity (MVPA). Two approaches were used to construct volume cutoffs for study purposes. One considered any amount of PA (absence vs presence), and the other classified participants into three categories according to volume in minutes of weekly PA: inactive (no PA); low active (up to 419); and high active (≥420). The highest cutoff (high active adolescents) and the classification of intensity of MVPA were adopted based on the recommendation of 60 min/day of PA at MVPA.^5^

The covariates were sex, age, parents’ level of education, work, alcohol, tobacco use, aggression, and study shift. The level of education of the head of the household was used as an indicator of socioeconomic status. Data for sex, age, and study shift were obtained through open questions. The other covariates were assessed using the following questions: Work status, “Do you work formally or informally in the period when you are not studying?”, with answer options “yes” or “no”; Alcohol use, “How often do you consume six or more doses at one time?”, “Never”, “Less than once a month”, “Monthly”, “Weekly”, and “Every or almost every day”, the cutoff adopted was any frequency of consumption of six or more doses; Tobacco use, “In the past 30 days, how many days have you smoked cigarettes?”, “None”, “1 or 2”, “3 to 5”, “6 to 9”, “10 to 19”, “20 to 29”, and “Every day”, any amount of tobacco use was considered as the cutoff; Aggression, “In the past 30 days, how many times have you been physically assaulted by an adult in your family?” or “In the past 30 days, how many times have you been involved in a fight where someone had a firearm or any kind of knife?”, “None”, “1”, “2 to 3”, “4 to 5”, “6 to 7”, “8 to 9”, “10 to 11”, and “12 or more”, and the cutoff adopted was ≥1.

Descriptive statistics were presented using absolute and relative frequency. The Pearson's chi-square test was adopted to assess the bivariate association between PA and covariates with mental health outcomes. Prevalence ratios and confidence intervals of 95% (CI95%) were estimated using Poisson regression considering the sampling procedures, using the command “svy” of the software Stata 14.0 (STATA Corp., College Station, Texas, United States). Covariates associated with each outcome which showed at least p<0.20 in the bivariate analysis were included simultaneously in the multivariate model. The statistical significance was set at p<0.05.

## RESULTS

Data collection was performed in 675 adolescents and the final sample was composed of 604 participants (22.8% of the study population) due to incomplete questions on any variable of the study. The distribution of the sample according to sociodemographic, covariate, and dependent variables of the study are described in Table 1.

**Table 1.** Characteristics of the 604 participants.

	n (%)
Sex	
Male	290 (48.0)
Female	314 (52.0)
Age (years)	
14–15	262 (43.4)
16	190 (31.5)
17–18	152 (25.2)
Parental educational level	
Elementary	212 (35.1)
High school	210 (34.8)
College	182 (30.1)
Aggression suffered	
No	551 (91.2)
Yes	53 (8.8)
Alcohol use	
No	413 (68.4)
Yes	191 (31.6)
Tobacco experience	
No	433 (71.7)
Yes	171 (28.3)
Work	
No	427 (70.7)
Yes	177 (29.3)
Suicidal ideation	
No	452 (74.8)
Yes	152 (25.2)
Suspicion of common mental disorders	
No	291 (48.2)
Yes	313 (51.8)
Negative self-perception	
No	377 (62.4)
Yes	227 (37.6)

The characteristics of the participants regarding PA are presented in Table 2. The prevalence of any amount of leisure PA practice varied from 31.2% at vigorous intensity to 44.4% at low intensity (sum of low active and high active prevalence). The prevalence of being high active (≥420 minutes/week) varied from 10.6% for low intensity to 16.2% for moderate intensity. The current guidelines of ≥420 minutes/week at MVPA were reached by 22% of the participants.

**Table 2.** Physical activity volume of the study participants according to intensity.

Volume (minutes/week)	Inactive^a^ n (%)	Low active^b^ n (%)	High active^c^ n (%)
LPA	336 (55.6)	204 (33.8)	64 (10.6)
MPA	339 (56.2)	167 (27.6)	98 (16.2)
VPA	416 (68.8)	123 (20.4)	65 (10.8)
MVPA	297 (49.2)	174 (28.8)	133 (22.0)

LPA: low intensity physical activity; MPA: moderate physical activity; VPA: vigorous physical activity; MVPA: moderate-to-vigorous physical activity

a Inactive: 0 minutes of physical activity in previous week; Low active

b Low active: up to 419 minutes/week of physical activity

c High active: ≥420 minutes/week of physical activity.

Bivariate and multivariate analyses of the association between any amount of PA according to intensity and mental health outcomes are described in Table 3. In the multivariate analysis, MVPA was associated with a lower prevalence ratio of suicidal ideation (PR 0.67), suspicion of common mental disorders (PR 0.77), and negative mental health self-perception (PR 0.68), all p<0.05. LPA was not associated with any of the outcomes (p>0.05).

**Table 3.** Association between any amount of physical activity according to intensity and mental health among adolescents.

	Suicidal ideation		Suspicion of commonmental disorders		Negative mentalhealth self-perception	
	%	Crude PR(95%CI)	%	Crude PR(95%CI)	%	Crude PR(95%CI)
LPA						
No	29.6	Reference	54.5	Reference	41.8	Reference
Yes	20.3	**0.70 (0.52–0.95)**	46.6	0.90 (0.77–1.06)	32.5	**0.76 (0.61–0.94)**
MVPA						
No	31.1	Reference	60.4	Reference	46.1	Reference
Yes	19.8	**0.58 (0.43–0.78)**	41.4	**0.62 (0.53–0.74)**	29.1	**0.58 (0.46–0.72)**
	**Adjusted PRa (95%CI)**		**Adjusted PRb (95%CI)**		**Adjusted PRc (95%CI)**	
LPA							
No	Reference		Reference		Reference	
Yes	0.80 (0.59–1.08)		0.97 (0.84–1.12)		0.81 (0.66–1.00)	
MVPA							
No	Reference		Reference		Reference	
Yes	**0.67 (0.49–0.92)**		**0.77 (0.66–0.91)**		**0.68 (0.54–0.86)**	

PR: prevalence ratio; CI: confidence interval; LPA: low intensity physical activity; MVPA: moderate-to-vigorous physical activity. In all cases, Pearson's chi-squared tests indicated p<0.05. Final multivariate models adjusted for covariates which showed at least p<0.20 in the bivariate analysis: ^a^sex, age, study shift, aggression experience, alcohol use, tobacco use, and work; ^b^sex, aggression experience, alcohol use, tobacco use and work; ^c^sex, study shift, alcohol and tobacco use. Bold denotes significance at p<0.05.

Table 4 describes the association between PA at different volumes and intensities with mental health outcomes. Multivariate analysis revealed that low active adolescents at MVPA have a lower likelihood of presenting suicidal ideation (PR 0.70), suspicion of common mental disorders (PR 0.76), and negative self-perception of mental health (PR 0.74) compared to those classified as inactive, all p<0.05. Similarly, high active adolescents presented a lower prevalence ratio for suicidal ideation (PR 0.62) and negative self-perception of mental health (PR 0.57), both p<0.05. No significant association was found for LPA in the multivariate analysis, all p>0.05.

**Table 4.** Association between physical activity according to volume and intensity with mental health among adolescents.

	Suicidal ideation		Suspicion of commonmental disorders		Negative mentalhealth self-perception	
	%	Crude PR (95%CI)	%	Crude PR (95%CI)	%	Crude PR (95%CI)
LPA						
Inactive^b^	29.6	Reference	54.5	Reference	41.8	Reference
Low active^b^	21.9	0.75 (0.54–1.05)	49.1	0.94 (0.79–1.11)	35.0	0.80 (0.64–1.02)
High active^b^	15.3	**0.52 (0.28–0.95)**	38.9	0.46 (0.55–1.04)	25.0	**0.57 (0.37–0.90)**
MVPA						
Inactive	31.1	Reference	60.4	Reference	46.1	Reference
Low active	22.0	**0.64 (0.45–0.91)**	46.2	**0.67 (0.55–0.82)**	34.4	**0.68 (0.53–0.87)**
High active	17.0	**0.50 (0.33–0.77)**	35.4	**0.55 (0.43–0.71)**	22.2	**0.43 (0.30-0.62)**
	Adjusted PR^d^ (95%CI)		Adjusted PR^b^ (95%CI)		Adjusted PR^b^ (95%CI)	
LPA							
Inactive	Reference		Reference		Reference	
Low active	0.86 (0.63–1.17)		0.99 (0.85–1.14)		0.83 (0.67–1.04)	
High active	0.53 (0.27–1.02)		0.90 (0.66–1.21)		0.70 (0.45–1.11)	
MVPA							
Inactive	Reference		Reference		Reference	
Low active	**0.70 (0.49–0.99)**		**0.76 (0.63–0.92)**		**0.74 (0.57–0.95)**	
High active	**0.62 (0.40–0.97)**		**0.79 (0.62–1.01)**		**0.57 (0.40–0.81)**	

PR: prevalence ratio; CI: confidence interval; LPA: low intensity physical activity; MVPA: moderate-to-vigorous physical activity.

a Inactive: 0 minutes of physical activity on last week; Low active

b up to 419 minutes/week of physical activity; High active

c ≥420 minutes/week of physical activity. Final multivariate models adjusted for covariates which showed at least p<0.20 in the bivariate analysis:

d sex, age, study shift, aggression experience, alcohol use, tobacco use, and work;

e sex, aggression experience, alcohol use, tobacco use and work;

f sex, study shift, alcohol and tobacco use. In all cases, Linear by Linear chi-squared tests indicated p<0.05. Bold denotes significance at p<0.05.

## DISCUSSION

The main findings of this study were that a better profile of mental health was described in adolescents who reported MVPA, independently of volume, while no association was found for LPA. Although the results varied according to the outcome, significant associations of MVPA were found for both the volume proposed by the current guidelines and for lower amounts of PA.

The results of the present study regarding MVPA corroborate previous information regarding the significant association between PA and a better mental health profile reported in the following studies: common mental disorders in a Brazilian sample,^9^ anxiety and depressive symptoms among adolescents from Canada,^6,7,10^ the United States of America (USA),^13^ England,^12^ Sweden,^11^ and suicidal ideation or attempt among adolescents from the USA,^13,14^ from other 48 countries,^15^ and from 52 low- and middle-income countries.^16^ However, for LPA the results are contrary, since no significant association was found for any outcome, independently of volume. Comparisons between studies are difficult due to heterogeneity regarding assessment and cutoffs adopted for both PA and mental health outcomes. However, the present study confirms the following aspects of the current recommendations of PA:^5^

a) PA is associated with mental health among adolescents;
b) PA is more relevant at higher intensities (MVPA); and
c) Some PA is better than none, considering that MVPA at any volume was associated with all variables analyzed. Furthermore, the results of the present study add information demonstrating that adolescents present mental health benefits in the three outcomes analyzed even when performing less than 60 min/day of MVPA,^5^ indicating that for mental health outcomes, different volumes of MVPA can be targeted.

Despite the association between PA and mental health described in the literature and corroborated by the present data, a relevant aspect to be discussed is the direction of the association, since most of the evidence is based on cross-sectional designs.^6,7,11,13,14,16^ Data from longitudinal studies indicate associations in both directions: baseline depressive symptoms predicting a decline in PA during adolescence^10^ and baseline low PA associated with future poor mental health.^7,12^ The impact of poor mental health on decreases in PA has been attributed to lower feelings of competence, autonomy, and relatedness, as well as a lack of energy, apathy, and social isolation.^10,24^ The effect of poor mental health on decreasing daily PA is pertinent because it deprives the adolescents of experiences of body movement and also exposes them to several other health-risks associated with insufficient PA.^5^ Likewise, several mechanisms support the belief that PA can improve mental health.^25^ PA can result in neuroplasticity, morphological adaptations in cerebral regions that are affected by depression, and an increase in neurotrophic factors in circulation. Another mechanism is the anti-inflammatory and antioxidant effect of PA, which is relevant since inflammation is associated with depression and treatment response. Furthermore, psychosocial mechanisms that emerge from socially active leisure time^24^ also play an important role in the prevention and treatment of common mental disorders by increasing autonomy, relatedness, self-esteem, self-competence, self-concept, self-efficacy, and social support.^8,25,26^

In the present study, only MVPA was associated with a lower prevalence of suicidal ideation, suspicion of common mental disorders, and negative self-perception of mental health. These results are in line with a systematic review that found positive effects of supervised aerobic-based MVPA for depression treatment.^27^ Generally, studies analyzed the association between PA and mental health and adopted a specific cutoff with fixed volume and intensity,^6,7,9,14-16^ which prevents knowledge of whether benefits can occur at different volumes and intensities. For this reason, the present study investigated the associations of different intensities and volumes of PA with mental health in adolescents, broadening the findings in the literature. Although there is information demonstrating an association when analyzing light intensity,^12^ the present study described an association only for MVPA, indicating that the benefits of PA on mental health outcomes are dependent on intensity. It is not possible to know which mechanisms explain why the associations occurred only for MVPA in the present study, because this information was not assessed, however, MVPA is commonly adopted in studies that investigate biological mechanisms underlying the association between exercise and mental health.^25^ Furthermore, psychosocial mechanisms may also be present, since sports are widely practiced and contribute greatly to MVPA among adolescents. These types of activity develop a broad range of competencies and psychosocial aspects that can contribute to the mental health of adolescents.^8,24^

Perceived mental health presented associations with different volumes of MVPA. Self-perception is a subjective measure of how people judge their health and is influenced by the knowledge of what mental health is and what influences it. In the sample analyzed, perceived mental health was associated with suspicion of common mental disorders (RP 2.27; 1.94–2.65) and suicidal ideation (RP 3.95; 2.89–5.38), which means that adolescents have a good perception about their mental health (data not shown). In general, affective responses are good during light intensity exercises, and decrease when vigorous intensity is imposed, although post exercise, the values are similar to those from light intensities.^28^ PA at higher intensities results in greater enjoyment due to elevated feelings of reward, excitement, and success.^29^ Another possible cause is the wide knowledge that PA is good for health, consequently, adolescents that perform PA at higher volumes and intensities naturally have better perceived mental health, as described by Zulyniak et al.^6^

The practical implications of the present results are that MVPA can aid the prevention and treatment of mental disorders among adolescents. However, the main factors for the emergence of mental disorders are widely described and include quality of life, relationships with peers and parents, living conditions, chronic disease, physical and sexual violence, discrimination, social stigma, lack of access to health service, forced marriages, orphanhood, and be part of minority groups.^1^ Another aspect is that the present results indicate that lower volumes of MVPA compared to the current guidelines were also associated with mental health. Besides, although adolescents should be encouraged to reach the recommendation since it is associated with a variety of health outcomes,^5^ mental health benefits can also be promoted by lower volumes of PA and it is relevant to motivate adolescents who are inactive.^19^ These findings are also relevant since the inactive adolescents also tended not to perceive the guidelines as reachable, resulting in lack of motivation to adhere to the recommendation of PA.^30^

Some limitations of this study should be mentioned. Since the data were cross-sectional, longitudinal associations or causal inferences between variables could not be verified and reverse causality cannot be disregarded. Although validated instruments were adopted to assess mental disorders, they only enable the identification of participants with a suspicion of mental disorder, but does not provide a diagnosis. PA was assessed using a self-report questionnaire and although it is widely used in epidemiological studies^18^ and presents acceptable validity, imprecision of estimates is still a concern. The sample size prevented stratification of the volume of PA into more groups due to the decrease in the power of the analyses, making the investigation of the volume of PA in lower intervals unfeasible. Conversely, the strengths of the present study are the probabilistic sample, validated instruments to assess exposures and outcomes, multivariate analysis adjusted for potential confounders, stratification of PA according to volume and intensity, and the addition of new information for outcomes that are determinant for the health and well-being of adolescents.

In conclusion, MVPA, regardless of volume, was associated with lower prevalence of suicidal ideation, suspicion of common mental disorders, and self-perception of mental health. No significant association was found for LPA. Although the achievement of guidelines should be encouraged, benefits to mental health also occurred with the performance of any volume of MVPA. These data can be used to motivate inactive adolescents to adopt an active lifestyle, even at lower levels than recommended for MVPA in the current guidelines.
